# Clinical significance of regional lymph node enlargement in patients with EGC within the expanded criteria for ESD

**DOI:** 10.1186/s12876-020-01197-z

**Published:** 2020-03-05

**Authors:** Dong Seok Lee, Jong Kyu Park, Sang Jin Lee, Gab Jin Cheon

**Affiliations:** 1 Department of Gastroenterology, SMG-SNU Boramae Medical Center, Seoul National University of College of Medicine, Seoul, South Korea; 2 Department of Gastroenterology, Gangneung Asan Hospital, University of Ulsan College of Medicine, Gangneung, South Korea

**Keywords:** Early gastric cancer, Lymph node enlargement, Endoscopic resection

## Abstract

**Background:**

Lymph node (LN) metastasis is negligible in early gastric cancer (EGC) within expanded criteria for endoscopic submucosal dissection (ESD). However, regional lymph nodes in abdominal CT scans are sometimes enlarged in patients with EGC within the expanded criteria for endoscopic submucosal dissection (ESD). In this study, we investigated the clinical significance of regional lymph node enlargement on abdominal CT scan in patients with EGC within the expanded criteria for ESD.

**Methods:**

From December 2010 to April 2015, among 301 patients with EGC within the ESD expanded criteria, 47 patients with regional lymph node enlargement shown by abdominal CT scan were prospectively enrolled. We performed surgical resection or periodic follow-up with abdominal CT scans and upper endoscopy every 6 months to evaluate whether the enlarged lymph nodes are due to metastasis or a reactive change.

**Results:**

The mean age of the 47 patients (38 males, 9 female) was 64.8 years. The enlarged lymph nodes were usually single (26/47, 44.6%) and sized as follows: 11 nodes were ≤ 5 mm, 19 were 6–10 mm, and 17 were ≥ 10 mm. Four of the 47 patients initially underwent surgical resection, and 8 patients underwent surgical resection after ESD. However, there was no lymph node metastasis in surgical specimens. Thirty-five patients received ESD and periodically followed up at a median duration of 56 months (IQR: 44–59 month). The enlarged lymph node disappeared in 12 of 35 patients, decreased in 9 patients and remained the same size in 13 patients, and increased in 1 patient.

**Conclusion:**

Regional lymph node enlargement on abdominal CT scan in patients within expanded criteria for ESD of ECG may be not due to metastasis but a reactive change.

## Background

Endoscopic resection is commonly used to treat early gastric cancer (EGC) without lymph node metastasis [1]. Endoscopic resection has a lower incidence of procedure-related mortality and morbidity and good long-term outcomes. Endoscopic resection is most commonly used instead of surgery in EGCs that meet the criteria for the expanded indication [2]. However, endoscopic resection for EGC has some limitations. The presence of lymph node metastasis around EGC cannot be evaluated by pathology. Some patients met the extended criteria before ESD, but they did not meet the expanded criteria on the final histopathologic result after ESD. These patients require additional surgery after ESD due to the possibility of lymph node metastasis, even though it was completely resected.

Gotoda et al. suggested that lymph node metastasis is negligible in patients with EGC within the expanded criteria [3]. However, regional lymph node enlargements are frequently discovered more than expected on peri-procedural abdominal CT scans, even though the patient is within the criteria. Various factors such as tumorous or non-tumorous disease can cause intra-abdominal lymph node enlargement. A differentiation between tumorous and non-tumorous disease is necessary. However, it is difficult to distinguish between reactive changes or gastric cancer metastasis on the basis of imaging studies without histologic evaluation. If lymph node enlargements on an abdominal CT are more than 1 cm and have a round shape, they may potentially be metastatic, and a surgical approach may be pursued for these lymph nodes even though the patients are within the expanded criteria. Accordingly, the aim of the present study was to investigate the clinical significance of regional lymph node enlargement on perioperative abdominal CT scan in patients with EGC within the expanded criteria.

## Methods

### Study population

We prospectively collected the clinical data of patients planned to receive ESD for EGC at Gangneung Asan Hospital in Gangneung, Korea from April 2010 to April 2015. Among 301 patients with EGC within the expanded criteria, 47 patients with regional lymph node enlargement on abdominal CT scans were recruited for this observational study. If EGC was diagnosed or suspected on the upper endoscopy and pathology, the patients underwent enhanced abdominal CT scan before ESD. If EGC was diagnosed after ESD in patient with adenoma or cellular atypia and abdominal CT was not checked, an abdominal CT scan was performed after ESD. We informed patients about the possibility of lymph node metastasis when the lymph nodes appeared enlarged on abdominal CT scans and decided on a treatment strategy such as surgical resection or careful follow up, although there was standard criteria of the treatment strategy. After providing informed consent, a clinical and demographic data were examined. The clinicopathologic characteristics included sex, treatment method, tumor size and location, tumor histology, ulcer presence, invasion depth, lympho-vascular invasion, lymph node size, lymph node number, lymph node location, lymph node status on CT, and follow up interval. This study was approved by the Institutional Review Board of the Gangneung Asan Hospital in Korea (institutional review board no. 4–2010-024), and all patients provided a written informed consent for the use of personal information in manuscripts. All patients provided informed consent before endoscopic examination. All patients who agreed to participate in this study were included in the study.

### Surveillance of lymph node enlargement

To evaluate the enlarged lymph nodes, regular periodic endoscopic examinations and contrast-enhanced abdominal-pelvic CT scans were performed in patient who want careful surveillance without additional surgical resection. Endoscopic examinations and abdominal CT scans were scheduled at 3 and at 6 months after ESD, then every 6 months for 3 years, and annually thereafter. We performed history taking and physical examination at every visit. For patients who underwent gastrectomy, enlarged lymph node was investigated to evaluate gastric cancer metastasis by pathologist.

### Definition of enlarged lymph nodes

Two radiologists were recruited to assess malignant potential the change in the internal architecture. Two radiologists were highly 10 years experienced in the diagnosis of stomach cancer. Generally, more than 1 cm of lymph nodes were regarded as a significantly enlarged lymph node. However, smaller than 1 cm of lymph nodes were classified as enlarged lymph nodes when the size was significantly increased compared to previous CT images. In addition to the change in size, the atypical features such as round shape with ill-defined margin, the long-to-short axis ratio decreases, eccentric cortical hypertrophy, central necrotic content, and heterogeneous enhancing pattern were regarded as a significantly enlarged lymph node. An area of low attenuation (10–18 HU) was considered to be evidence of nodal necrosis.

### ESD for EGC

Thirty-five patients underwent ESD procedures. A standard single channel endoscope (GIF-Q260J or GIF-H260Z; Olympus Co. Ltd) was used for all ESD procedures. Electrocautery (VIO 300D; ERBE, Tübingen, Germany) with an argon plasma coagulation (Olympus Co. Ltd) was used to mark gastric lesions. A hook knife (Olympus Co. Ltd) and an insulation tipped knife 2 (Olympus Co. Ltd) were used for circumferential incision and submucosal dissection. All ESD procedures were performed by expert gastroenterologists. The following criteria must be met for curative endoscopic resection for tumors of expanded indication when they are not accompanied with lymphovascular infiltration [ly(−), v(−)]: (1) Any tumor size, without ulceration, histologically the differentiated type, pT1a; (2) tumor size ≤3 cm, with ulceration, histologically the differentiated type, pT1a; (3) tumor size ≤2 cm, without ulceration, histologically the undifferentiated type, pT1a; (4) tumor size < 3 cm, histologically the differentiated type, pT1b (SM1, < 500 um from the muscularis mucosa) [4]. If any of the above criteria is not met, it is classified as non-curative resection [4]. Additional treatment was conducted according to curative resection. Additional gastrectomy was performed for patients in case of non-curative resection [5]. After ESD, specimen was fixed on a polystyrene plate and dipped in 8% formaldehyde for 24 h. According to the recommendations of Japanese Classification of Gastric Carcinoma, fixed specimens were cut into 2 mm thick sections and then stained with hematoxylin and eosin [6]. All slides from specimens were evaluated by the two pathologists to confirm that the entire lesion of gastric cancer had been removed [7]. Histological diagnosis of the specimen was made according to the Vienna classification of gastrointestinal epithelial tumors [8].

### Surgical resection

Although all patients were within the expanded criteria for ESD for EGC, if the patients had lymph node enlargement on their abdominal CT scan and wanted surgical resection, they underwent distal or total gastrectomy with lymph node dissection by experienced surgeons. The extent of resection depended on the tumor location and extent. Lymphadenectomy was performed based on the rules of the Japanese Research Society for Gastric Cancer. To evaluate the curative resection (R0), macroscopic or microscopic examination was performed.

### Additional surgical resection after ESD

If EGC was diagnosed after ESD in patients with adenoma or cellular atypia and abdominal CT was not checked, an abdominal CT scan was performed after ESD. When lymph node enlargement showed on the CT scan after ESD, additional surgical resection was performed in cases when the patient wanted it, although the lesions were within the expanded criteria.

### Statistical analysis

Data were presented as the median and interquartile range (IQR). Logistic regression analyses were performed to identify the variables independently associated with lymph node regression (decreased or disappeared lymph node). *P* < .05 was considered statistically significant. Data were analyzed with SPSS version 23 software (SPSS Inc., Chicago, Ill).

## Results

### Baseline patient characteristics

Forty-seven patients (38 men and 9 women; median age: 64 years; IQR: 58.5–72.5 years) within the expanded criteria of ESD for EGC and with lymph node enlargement on an abdominal CT scan were enrolled in this study. Patient and clinicopathologic characteristics are shown in Table 1. The enlarged lymph nodes were most commonly just a single lymph node (26/47, 66.0%). The size of the lymph node was ≤5 mm in 11 patients, 6–10 mm in 19 patients, and ≥ 10 mm in 17 patients. The shape of the enlarged lymph node was oval in 27 patients and round in 20 patients. Of the 47 enrolled patients, 4 initially underwent surgical resection, 8 underwent surgical resection of the enlarged lymph node after curative ESD, and 35 received ESD.

**Table 1:** Clinicopathologic characteristics of the study population (*N* = 47)

Characteristics	Number of Patients (%)
Sex	
Male	38
Female	9
Treatment method	
Distal Gastrectomy	4
ESD + DG	8
ESD	35
Tumor location	
Upper body	6
Middle body	11
Lower body	39
Tumor size (mm)	
< 10 mm	14
< 20 mm	25
< 30 mm	7
≥ 31 mm	1
Histologic type	
Differentiated	46
Undifferentiated	1
Ulcer	
Absence	43
Presence	4
Lympho-vascular invasion	
Absence	47
Presence	0
Tumor invasion	
Mucosa	40
submucosa	7
Size of lymph node	
≤ 5 mm	11
6 mm ≤ lymph node < 10 mm	19
≥ 10 mm	17
Number of lymph node	
Single	26
Multiple	21
Shape of lymph node	
Round	20
Oval	27
Location of lymph node	
Peri-gastric	37
Gastro hepatic	7
Hepatoduodenal ligament	1
Peri-pancreatic	1
Peri-aortic	4
Cardiophrenic	1
Porta hepatitis	1
Duration of follow up (35 patients treated with ESD)	
< 4 year	12
< 5 year	15
< 6 years	3
≥ 6 years	5

*ESD* Endoscopic submucosal dissection

### Clinical significance of enlarged lymph node within the expanded criteria

**Fig. 1:**
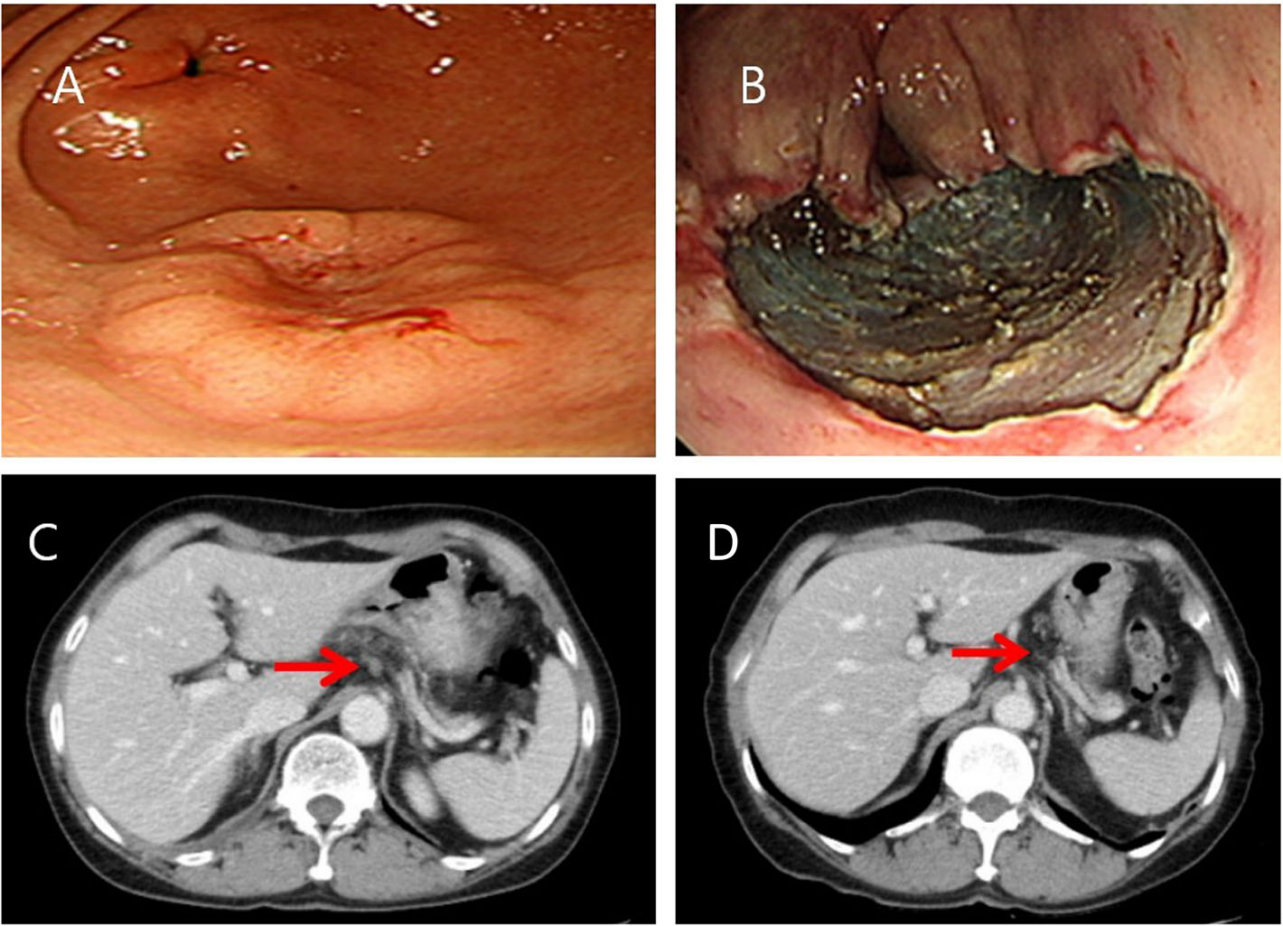
**a**, 2-cm sized superficial elevated lesion with central depression (0-IIa and 0-IIc) on the greater curvature of the distal antrum. **b**, Post ESD ulceration. (Well differentiated adenocarcinoma invaded into 20 μm of submucosal layer from muscularis mucosa without involvement of the lateral and deep margins on histopathological examination) **c**, Enlarged perigastric lymph node (arrow, diameter, approximately 10 mm) on contrast-enhanced abdominal CT scan. **d**, No visible lymph node (arrow) on follow up abdominal CT scan after 12 months

Among the 12 patients who underwent surgical resection, there was no lymph node metastasis in the surgical specimens. Thirty-five patients received only ESD and followed up periodically for a median duration of 56 months (range: 44–59 months). The enlarged lymph nodes disappeared in 12 patients (Fig. 1), decreased in 9 patients and remained the same size in 13 patients, and increased in 1 patient (Table 2). The median duration to disappearance of enlarged lymph node were 15 months (range: 4-60 months). In 7 cases of abdominal CT scan was performed after ESD, enlarged lymph node was disappeared in two cases, decreased in 1 case and remained in 4 cases. In the disappeared cases, enlarged lymph node was decreased at 3 months and disappeared at 6 months after ESD in one case and disappeared at 28 months in another case. In the case of an increased lymph node during the follow-up period, the primary tumor was measured 1.0 × 0.6 cm and was classified as well-differentiated mucosal cancer (M3) without ulceration, which was resected curatively with negative resection margin; 5 mm and 8 mm sized lymph nodes were initially located in right perigastric area. During a 5-year follow-up period, the 8 mm sized lymph node disappeared, but the 5 mm sized lymph node increased to 9 mm and became more intense. Because the 80-year-old patient refused the surgical resection, we could not further evaluate the increased lymph node.

**Table 2:** Change in enlarged lymph nodes during follow up after ESD (*n* = 35)

Follow up duration (median, IQR)	56 months (IQR, 44–59)
Change of lymph node	Number
No interval change	13
Decreased	9
Disappeared	12
Increased	1

**Fig. 2:**
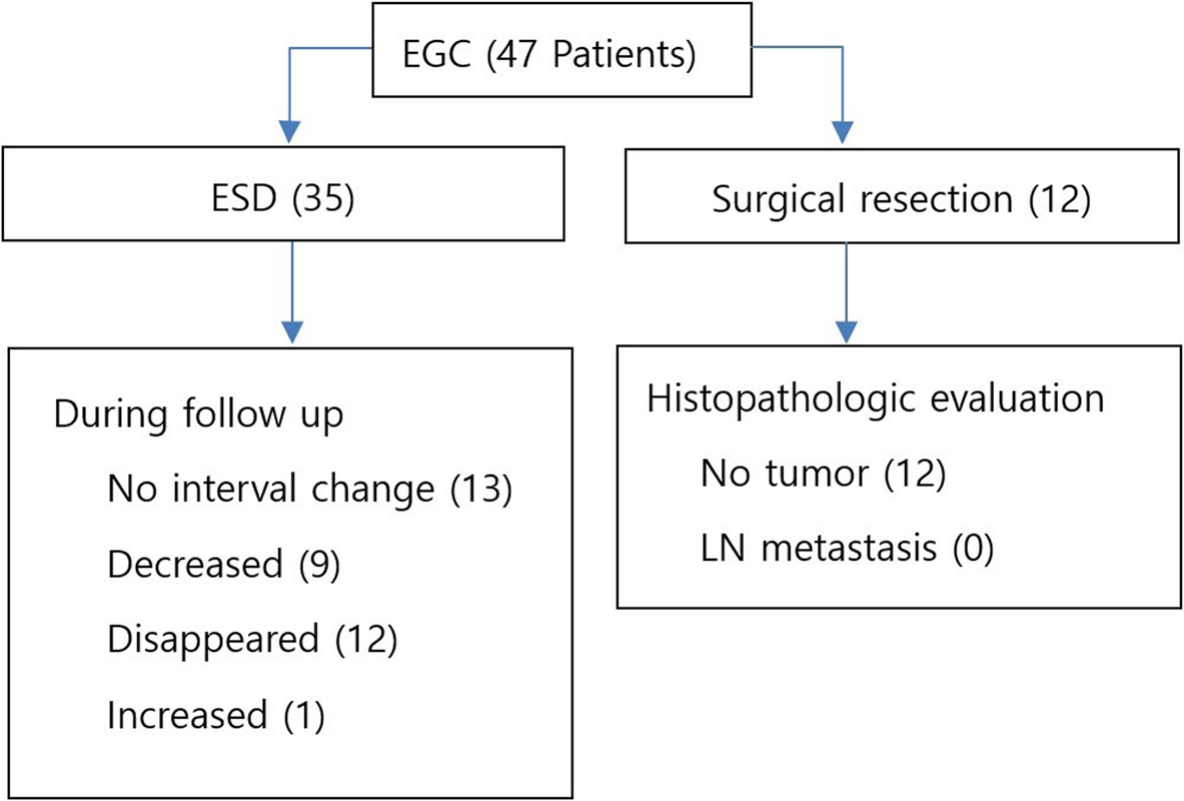
Flowchart of patients included in this study: 47 patients were included in outcomes. EGC, early gastric cancer; ESD, endoscopic submucosal dissection

Seventeen patients of 47 patients had enlarged lymph nodes with a round shaped more than 1 cm in size, suggestive of lymph node metastasis. Of those 17 patients, 5 patients underwent surgery and found that the enlarged lymph nodes were reactive changes in pathology. Of those 17 patients, 12 patients followed up regularly with CT scans and EGD for a median of 51 months. The enlarged lymph node disappeared in 2 cases, decreased in size in 4 case, and experienced no change in size in 6 cases. The outcomes for all patients are shown in Fig. 2.

Metachronous gastric cancer were detected in 5 (14.3%) out of 35 patient who were follow up periodically after ESD. All of them were successfully treated with repeated ESD.

In multivariate analysis to identify the variables associated with lymph node regression (decreased or disappeared lymph node), multiple lymph node enlargement three and more was associated with lymph node regression. But the clinical features of primary tumor, size and shape, define of margin, fat hilum of enlarged lymph node was not associated (Table 3).

**Table 3:** Multivariate logistic regression analysis of variables associated with lymph node regression

Variables	Odds ratio	95% confidence interval	*P* value
Age	0.997	0.923–1.077	0.947
Sex (Female)	1.516	0.071–32.261	0.790
Tumor size (> 2 cm)	1.333	0.178–9.987	0.780
Tumor location			0.996
upper	1		
mid	0.885	0.064–12.268	0.927
lower	0.985	0.033–29.047	0.993
Undifferentiated cancer	10.104	0.787–129.662	0.076
Submucosal invasion	1.225	0.627–8.659	0.994
Ulceration	0.259	0.001–51.089	0.616
Status of LN			
Round shape	0.995	0.127–7.814	0.996
≥ 1 cm size	0.221	0.024–2.054	0.184
Multiple (≥3)	0.106	0.013–0.929	0.043
Ill-defined margin	0.333	0.010–9.925	0.491
Fat hilum	2.228	0.080–61.817	0.637
Regional LN	0.318	0.018–5.497	0.431

## Discussion

The incidence of EGC is extremely high in Asian countries and ESD is commonly used for the endoscopic treatment of EGC [2]. ESD removes only malignant lesions without removing lymph nodes. ESD is only indicated EGC patients without lymph node metastasis. In Japanese EGC treatment guidelines, lymph node metastasis is considered negligible within the expanded criteria of ESD for EGC. Therefore, EGC within the expanded criteria after ESD is considered a curative resection [3]. Some patients met the extended criteria before ESD, but they did not meet the expanded criteria on the final histopathologic result after ESD. These patients require additional surgery after ESD [9]. In a recent study, the curative resection rate was reported to be 81.9% in patients who underwent ESD for EGC [10]. Lymph node enlargement is sometimes observed in peri-procedural abdominal CT scans even in patients within the expanded criteria. In that case, it is difficult to decide whether to perform ESD or surgical resection because the physician cannot completely rule out the possibility of lymph node metastasis.

With the development of multi-detector computed tomography, the incidental detection of lymph node enlargement has increased on the abdomen. Common causes of enlarged lymph nodes in the abdomen are malignant disease and reactive changes caused by infection or inflammation. Infectious diseases due to bacteria or viruses, such as tuberculosis and inflammatory responses such as appendicitis and diverticulitis, can cause intra-abdominal lymph node enlargement. Metastatic malignancies from gastric cancer or solid or hematopoietic tumors may also produce abdominal lymph node enlargement [11]. Differentiation between malignant and reactive lymph node enlargement is essential for the treatment of EGC.

Although it is difficult to distinguish benign lymph nodes from malignant lymph nodes in CT, abnormal features of malignant nodes are greater than 1 cm in size, are rounded in shape, have eccentric cortical hypertrophy, and are increased in number (a cluster of ≥3 lymph nodes in a single nodal station, or a cluster of ≥2 lymph nodes in ≥2 regions). Benign features are a size less than 1 cm, an oval or elongated shape, and a central fatty hilum [3, 12]. However, 1 cm size is not an absolute reference to assess malignant lymph nodes because small metastases in normal-sized lymph nodes are quite common [13].

In our study, there were 17 cases of abnormal lymph nodes greater than 1 cm in size and of a round shape. However, there was no increase in the size or number of nodes after follow up, and no lymph node metastasis was identified with post-surgical pathology. Rather, 5 mm sized small lymph node was increased during follow-up although it was not possible to confirm the metastasis. Almost cases of abnormal features were found to be simple reactive changes after surgical resection or follow-up. Therefore, EGC within the expanded criteria can be carefully observed, although there were two or more of these abnormal features in the enlarged lymph nodes.

In our study, multiple lymph node enlargement three and more was associated with lymph node regression in multivariate analysis. These results are consistent with the finding that multiple lymph node enlargement is more commonly seen in non-malignant diseases such as inflammation or infection, unless lymphoma or advanced gastrointestinal malignancy [11]. Therefore, closed surveillance can be recommended rather than surgical resection in patient with multiple lymph node enlargement three and more.

In a previous study, local recurrences post-ESD were found after a median follow-up time of 48 months [14]. In this study, there were no lymph node metastasis for 56-months of follow-up, which was a long enough duration to evaluate the enlarged lymph nodes. We therefore conclude that each lymph node enlargement might be a reactive change and not metastasis.

In this study, metachronous gastric cancer were detected in 5 (14.3%) of the periodically followed up patients after ESD. The incidence rate of metachronous gastric cancer after endoscopic resection is 4.9–9.1% [2, 15–17]., which is higher than that after surgery due to preservation of the entire stomach. Therefore, careful EGD surveillance instead of abdominal CT surveillance is important to detect metachronous cancer at an early stage in patients within expanded criteria. In our study, the incidence of metachronous cancer was more than that reported in long-term studies. The exact reason for this is unknown, but the enlargement of perigastric lymph nodes may reflect severe gastric inflammation. Therefore, patients with lymph node enlargement such as those in this study may require stricter EGD and abdominal CT surveillance after ESD.

Gotoda et al. suggested that lymph node metastasis can be negligible in patients with EGC within the expanded criteria [3]. Recently, Hasuike et al. revealed that patients with expanded criteria in UL-negative tumors measuring > 2 cm or UL-positive tumors measuring < 3 cm are acceptable for ESD and should receive standard treatment instead of gastrectomy [18]. However, all EGCs within the expanded criteria may not have the same risk for lymph node metastasis. One study reported lymph node metastasis in three patients (2.3%) after surgery in a group of 181 patients who met the expended criteria [19]. Therefore, the possibility of lymph node metastasis is not completely excluded even within the criteria. Especially when the lymph node is enlarged in CT, the possibility of metastasis should be considered. In the above study, they did not show whether the lymph node enlargement appeared on pre-operative abdominal CT scans in the cases with lymph node metastasis [19]. Because lymph node metastasis found accidentally in post-surgical pathology and lymph node enlargement found on peri-ESD abdominal CT are different, it is very important to know the clinical significance of lymph node enlargement observed on peri-ESD abdominal CT scans. Lymph node enlargement was seen in 47 (15.6%) of 301 patients satisfying the expanded criteria, which is more commonly than expected. Our study is therefore meaningful and helpful for establishing treatment strategies.

This study has some limitations. First, our study included small-sized enlarged lymph nodes less than 5 mm. Although more than 10 mm of lymph node is generally considered lymph node metastasis, potential of metastasis of EGC cannot be excluded in small-sized lymph node with atypical features or increased size compare with precious CT scan. Second, we did not randomize to surgery or periodic follow-up. Because our purpose to evaluate the clinical significance of lymph node enlargement in patient of EGC within expanded criteria, we could conclude that the enlarged lymph node was a reactive change irrespective of surgery or periodic follow-up. Third, our study included some cases of enlarged lymph node on abdominal CT scan after ESD. Although post-ESD ulcer also cause a lymph node enlargement, only one case of lymph node disappearing at 6 months after ESD is suspected and the rest is not related to post-ESD ulcer. Fourth, small sample sized single center study design might have an inherent selection bias. Fifth, the flow of research can show observational nature. Despite these limitations, this study provides useful information for making a decision about lymph node enlargement observed with abdominal CT. And this study was performed prospectively, followed patients for a long term, and was the first observation to clarify the treatment strategy before the procedure.

## Conclusions

Lymph node enlargement on abdominal CT scans in patients with EGC within the expanded criteria was negligible and might not associated with gastric cancer metastasis, especially in patient with multiple lymph node enlargement three and more. On the basis of this favorable long-term outcomes, ESD may be appropriate for EGC within the expanded criteria even though regional lymph nodes are enlarged on peri-procedural abdominal CT scans.

## Data Availability

The datasets used and/or analyzed during the current study available from the corresponding author on reasonable request.

## Abbreviations

CI: Confidence interval
CT: Computed tomography
EGC: Early gastric cancer
ESD: Endoscopic submucosal dissection
SM: Submucosal

## References

[1] Soetikno R, Kaltenbach T, Yeh R, Gotoda T (2005). Endoscopic mucosal resection for early cancers of the upper gastrointestinal tract. J Clin Oncol.

[2] Kim YI, Kim YW, Choi IJ, Kim CG, Lee JY, Cho SJ, Eom BW, Yoon HM, Ryu KW, Kook MC (2015). Long-term survival after endoscopic resection versus surgery in early gastric cancers. Endoscopy.

[3] Gotoda T, Yanagisawa A, Sasako M, Ono H, Nakanishi Y, Shimoda T, Kato Y (2000). Incidence of lymph node metastasis from early gastric cancer: estimation with a large number of cases at two large centers. Gastric cancer : official journal of the International Gastric Cancer Association and the Japanese Gastric Cancer Association.

[4] Japanese Gastric Cancer Association: Japanese gastric cancer treatment guidelines 2014 (2017). ver. 4. Gastric Cancer.

[5] Ryu KW, Choi IJ, Doh YW, Kook M-C, Kim CG, Park H-J, Lee JH, Lee J-S, Lee JY, Kim YW (2007). Surgical indication for non-curative endoscopic resection in early gastric Cancer. Ann Surg Oncol.

[6] Japanese Gastric Cancer Association. Japanese classification of gastric carcinoma: 3rd English edition. Gastric Cancer. 2011;14(2):101-12. 10.1007/s10120-011-0041-5 21573743

[7] Yang MJ, Shin SJ, Lee KS, Lee KM, Lim SG, Kang JK, Hwang JC, Kim SS, Lee D, Kim JS (2015). Non-neoplastic pathology results after endoscopic submucosal dissection for gastric epithelial dysplasia or early gastric cancer. Endoscopy.

[8] Suzuki H, Oda I, Abe S, Sekiguchi M, Mori G, Nonaka S, Yoshinaga S, Saito Y (2016). High rate of 5-year survival among patients with early gastric cancer undergoing curative endoscopic submucosal dissection. Gastric cancer : Official J Int Gastric Cancer Assoc Japanese Gastric Cancer Assoc.

[9] Kim EH, Park JC, Song IJ, Kim YJ, Joh DH, Hahn KY, Lee YK, Kim HY, Chung H, Shin SK (2017). Prediction model for non-curative resection of endoscopic submucosal dissection in patients with early gastric cancer. Gastrointest Endosc.

[10] Min BH, Kim ER, Kim KM, Park CK, Lee JH, Rhee PL, Kim JJ (2015). Surveillance strategy based on the incidence and patterns of recurrence after curative endoscopic submucosal dissection for early gastric cancer. Endoscopy.

[11] Lucey BC, Stuhlfaut JW, Soto JA (2005). Mesenteric lymph nodes seen at imaging: causes and significance. Radiographics : Rev Publ Radiol Soc North Am, Inc.

[12] Torabi M, Aquino SL, Harisinghani MG (2004). Current concepts in lymph node imaging. J Nucl Med.

[13] Epstein JI, Oesterling JE, Eggleston JC, Walsh PC (1986). Frozen section detection of lymph node metastases in prostatic carcinoma: accuracy in grossly uninvolved pelvic lymphadenectomy specimens. J Urol.

[14] Lee JY, Choi IJ, Cho SJ, Kim CG, Kook MC, Lee JH, Ryu KW, Kim YW (2012). Routine follow-up biopsies after complete endoscopic resection for early gastric Cancer may be unnecessary. J Gastric Cancer.

[15] Jeon HK, Kim GH, Lee BE, Park DY, Song GA, Kim DH, Jeon TY (2018). Long-term outcome of endoscopic submucosal dissection is comparable to that of surgery for early gastric cancer: a propensity-matched analysis. Gastric Cancer : Official J Int Gastric Cancer Assoc Japanese Gastric Cancer Assoc.

[16] Pyo JH, Lee H, Min BH, Lee JH, Choi MG, Lee JH, Sohn TS, Bae JM, Kim KM, Ahn JH (2016). Long-term outcome of endoscopic resection vs. surgery for early gastric Cancer: a non-inferiority-matched cohort study. Am J Gastroenterol.

[17] Cho JH, Cha SW, Kim HG, Lee TH, Cho JY, Ko WJ, Jin SY, Park S (2016). Long-term outcomes of endoscopic submucosal dissection for early gastric cancer: a comparison study to surgery using propensity score-matched analysis. Surg Endosc.

[18] Hasuike N, Ono H, Boku N, Mizusawa J, Takizawa K, Fukuda H, Oda I, Doyama H, Kaneko K, Hori S (2018). A non-randomized confirmatory trial of an expanded indication for endoscopic submucosal dissection for intestinal-type gastric cancer (cT1a): the Japan clinical oncology group study (JCOG0607). Gastric Cancer : Official J Int Gastric Cancer Assoc Japanese Gastric Cancer Assoc.

[19] Jee YS, Hwang SH, Rao J, Park DJ, Kim HH, Lee HJ, Yang HK, Lee KU (2009). Safety of extended endoscopic mucosal resection and endoscopic submucosal dissection following the Japanese gastric Cancer association treatment guidelines. Br J Surg.

[20] Shin SY, Park JK, Lee SJ, Cheon GJ. Clinical significance of regional lymph nodes enlargement in patient with EGC within expanded criteria. Gastroenterol Hepatol. 2016;31(S3):Abstract #1131. 10.1186/s12876-020-01197-z PMC7059300 32138692

